# Is TikTok Addiction Associated with Substance Use and Compulsive Buying Among University Students in Tunisia? A National Cross-Sectional Study

**DOI:** 10.1192/j.eurpsy.2026.11314

**Published:** 2026-07-31

**Authors:** S. Ben Fredj, R. Ghammam, N. Zammit, A. Skhiri, O. Ben Saad, A. Chouchen, I. Harrabi

**Affiliations:** 1 Department of Preventive Medicine and Epidemiology, “LR19SP03”, University Hospital Farhat Hached; 2 Faculty of Medicine of Sousse, University of Sousse; 3 Department of Occupational Medicine, “LR19SP03”, University Hospital Farhat Hached, Sousse, Tunisia

## Abstract

**Introduction:**

The addictive design of social media platforms, particularly TikTok’s algorithmically-driven short-form video feed, poses a significant risk for the development of problematic use patterns among young adults. TikTok addiction, characterized by a loss of control and pre-occupation with the platform, is increasingly recognized as a behavioral disorder with serious consequences. The specific association between TikTok addiction, substance use, and compulsive buying remains underexplored, particularly in the Arab world. Investigating this triad is crucial to understanding the broader public health impact of TikTok addiction on student well-being.

**Objectives:**

This study aimed to assess the relationship between TikTok addiction, lifestyle habits, substance use (tobacco and alcohol), and compulsive buying behavior among Tunisian university students.

**Methods:**

We conducted a cross-sectional online study among university students in Tunisia during the academic year 2023-2024. The call for participation was posted on social media. Population consisted of university students enrolled in public and private institutions during the 2023-2024 academic year. The survey questionnaire was made available in the Arabic language. TikTok addiction were assessed utilizing the Arabic adaptation and validation of the six-point modified Bergen Facebook Addiction Scale. The statistical analyses were conducted using the Statistical Package for Social Sciences (SPSS version 20).

**Results:**

A total of 326 university students were included. The majority of participants (71.5%) were female and 57% were undergraduates. the average age was 23.06±3.03 years. The prevalence of TikTok addiction was 10.1% and 28.2% among TikTok users. Females were more addicted to TikTok than males (11.2% vs. 7.5% respectively; p=0.014). Students who use tobacco and/or alcohol shown a significant higher tendency towards TikTok addiction compared to non-users. Students who reported not adhering to recommended levels of physical activity and healthy eating displayed a higher prevalence of TikTok addiction. Conversely, a lower prevalence was observed among those who engaged in leisure activities more frequently. However, no significant association was found

**Conclusions:**

These results suggest that these behaviors may share common underlying mechanisms, such as impulsivity or dysregulated reward processing. Consequently, public health initiatives should not address TikTok addiction in isolation but consider it as part of a potential cluster of risky behaviors. Future longitudinal research is needed to elucidate the causal pathways directing these relationships.

**Disclosure of Interest:**

None Declared

